# COVID-19 Vaccination Rates and Predictors of Vaccine Uptake Among Adults With Chronic Obstructive Pulmonary Disease: Insights From the 2022 National Health Interview Survey

**DOI:** 10.7759/cureus.59230

**Published:** 2024-04-28

**Authors:** Oyinlola O Fasehun, Oluwafeyi Adedoyin, Charity Iheagwara, Ifeyinwa H Ofuase-Lasekan, Sapana Manandhar, Natalie A Akoto, Taiwo Ajani, Chuka G Nwume, Joshua T Green, Okelue E Okobi

**Affiliations:** 1 Internal Medicine, University of Texas Rio Grande Valley Knapp Medical Center, Weslaco, USA; 2 Internal Medicine, Access Family Clinic, Joplin, USA; 3 Infectious Diseases, Saint Michael's Medical Center, Newark, USA; 4 Family Medicine, Provincial Health Services Authority, Vancouver, CAN; 5 Pediatric Medicine, Jiamusi University, Covington, USA; 6 Epidemiology, Emory University Rollins School of Public Health, Atlanta, USA; 7 Internal Medicine, Obafemi Awolowo University, Ile-Ife, NGA; 8 Family Medicine, University of Port Harcourt, Port Harcourt, NGA; 9 Surgery, Sibley Memorial Hospital, Washington, DC, USA; 10 Family Medicine, Larkin Community Hospital Palm Springs Campus, Miami, USA; 11 Family Medicine, Medficient Health Systems, Laurel, USA; 12 Family Medicine, Lakeside Medical Center, Belle Glade, USA

**Keywords:** age, covid-19, prevention, vaccine, chronic pulmonary disease

## Abstract

Background and objective

The coronavirus disease 2019 (COVID-19) vaccination rates and predictors of vaccine uptake among patients with chronic obstructive pulmonary disease (COPD) in the United States are unknown. In light of this, we assessed COVID-19 vaccination rates in this population and evaluated predictors of vaccine uptake.

Methods

Using 2022 survey data from the National Health Interview Survey (NHIS), 1486 adults with COPD who responded with "yes/no" to whether they had received the COVID-19 vaccine were identified, including those who had received booster doses. A chi-square test was used to ascertain differences between those who had received the vaccine and those who had not, as well as between those who had received booster doses and those who had not. A logistic regression was used to evaluate predictors of COVID-19 vaccination uptake.

Results

A total of 1195 individuals among 1486 respondents with chronic pulmonary disease (78.4%) had been vaccinated against COVID-19, and 789/1195 (62.5%) had received booster shots. The majority of individuals were aged 65 years and above, exceeded the 1+ threshold for the ratio of family income to poverty (RFIP), and were covered by insurance. Positive predictors of COVID-19 vaccination were as follows: age 40 - 64 years (OR: 2.34, 95% CI: 1.31 - 4.19; p=0.004) and 65 years and above (OR: 1.93, 95% CI: 1.36 - 2.72; p<0.001), RFIP threshold of ≥1 (OR: 2.02, 95% CI: 1.42 - 2.88; p<0.001), having a college degree (OR: 1.92, 95% CI: 1.92 - 3.26, p=0.016), and being insured (OR: 3.12, 95% CI: 1.46 - 6.66, p=0.003). The current smoking habit negatively predicted the uptake (OR: 0.54, 95% CI: 0.33 - 0.87, p=0.012). The positive predictors of COVID-19 vaccination boosters were as follows: age 40 - 64 years (OR: 2.72, 95% CI: 1.39 - 5.30, p=0.003) and 65 years and above (OR: 4.85, 95% CI: 2.45 - 9.58, p<0.001). Being from the non-Hispanic (NH) black ethnicity negatively predicted receiving the COVID-19 booster (OR: 0.55, 95% CI: 0.36 - 0.85, p=0.007).

Conclusions

While COVID-19 vaccination rates are fairly satisfactory in COPD patients, the uptake of booster vaccines is relatively lower in this population. Socioeconomic and behavioral factors are associated with poor vaccine uptake, and targeted interventions should be implemented to address these factors.

## Introduction

In the United States (US), the coronavirus disease 2019 (COVID-19) pandemic resulted in 6.62 million quality-adjusted life-years lost till March 13, 2021 [1]. Racial minorities and those with medical comorbidities have borne the brunt of the ravages of the pandemic [1], particularly in populations with chronic respiratory diseases [2]. Associated with this high mortality has been the heavy burden of the disease on healthcare costs, with billions of US dollars spent on patient care and lost due to lost productivity days [3]. Several public health interventions were implemented to address the crippling effects of the pandemic, including the use of personal protective equipment, screening to detect early disease cases, contact tracing, and suppression/containment [4]. The first two interventions had positive results with a significant effect in slowing the spread of disease and changing the mortality and morbidity trend [5,6], while the latter had mixed results [7].

In addition to the above measures, various vaccines were also introduced, and booster shots were later recommended. Unfortunately, this was met with mixed reactions in various patient populations, with the decades-long problem of vaccine hesitancy resurfacing in the wake of this pandemic [8,9]. For example, a cross-sectional study involving patients with chronic lung disease revealed that previously receiving the influenza vaccine, having no history of cigarette use, and expressing trust in vaccine development were identified as positive predictors for COVID-19 vaccine acceptance. Conversely, younger age and lower levels of education were identified as negative predictors [10].

There is limited understanding regarding COVID-19 vaccination rates among patients with chronic obstructive pulmonary disease (COPD), a subset of chronic lung diseases in the US; data on factors predicting COVID-19 vaccine uptake in this population are scarce as well. In this study, we set out to assess the uptake of COVID-19 vaccines among patients with COPD and evaluate the factors predictive of vaccine uptake in this population. We believe such a research effort holds great significance as COVID-19 vaccination has been shown to be protective via the stimulation of systemic and airway immune responses in this population [11-12], thereby generally reducing adverse health outcomes.

## Materials and methods

Study design

This cross-sectional study utilized sample adult data from the publicly available 2022 National Health Interview Survey (NHIS). The NHIS is the principal source of information on the health of the noninstitutionalized civilian population in the US. It is conducted throughout the year by the National Center for Health Statistics (NCHS). The main objective of collecting this data is to monitor the health status of the US population through data analysis of a broad range of health topics such as vaccination status and medical comorbidities. Participants are typically interviewed face-to-face, but follow-up interviews may be conducted over the telephone. Further information about this NHIS data is available online [13]. Institutional review board approval was not required for this study as it involved publicly available and de-identified data.

Study population

**Figure 1:**
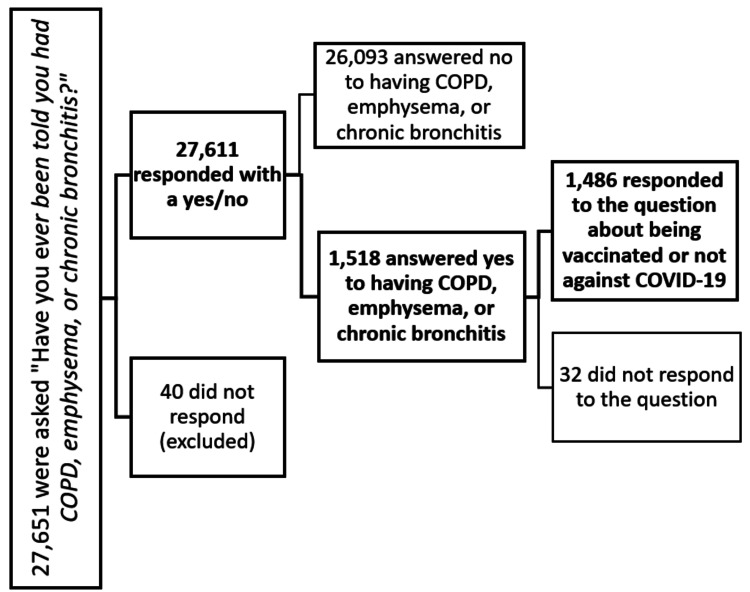
Chart depicting sample selection for the study COPD: chronic obstructive pulmonary disease; COVID-19: coronavirus disease 2019

We contacted 27,651 individuals who were asked the question “Have you ever been told that you had COPD, emphysema, or chronic bronchitis ?”. Of this, 27,611 responded either yes or no, while 40 did not respond. These non-responders, making up about 0.1% of the population, were excluded from the study; 1518/27,611 respondents reported having COPD, of whom only 1486 responded to the question about whether they were vaccinated against COVID-19, as shown in Figure 1 below, and were used as the sample population.

Sociodemographic variables

The sociodemographic variables assessed were as follows: age (<40, 40 - 64, and ≥65 years), ethnicity [non-Hispanic (NH) white, Hispanic, NH black, and NH others], gender (male and female), level of education (graduated from college/did not graduate from college), the ratio of family income to poverty (RFIP) (<1 and ≥1), and insurance status (uninsured and insured).

Comorbidities

Respondents were asked about their medical comorbidities - which were classified as follows: diabetes mellitus (have diabetes mellitus/no diabetes mellitus), hypertension (have hypertension/no hypertension) - and BMI (obese/not obese). BMI was measured during the survey data collection. The respondents were also classified based on the following parameters: smoking status (never smokers, current smokers, and former smokers); chronic kidney disease (CKD) status (having CKD/no CKD), and COPD (having COPD/no COPD).

COVID-19 vaccination

Respondents were asked if they had been vaccinated against COVID-19, and they responded with a yes/no. Those who responded affirmatively were then asked how often they had been vaccinated against COVID-19. The available responses were as follows: 1, 2, 3, and 4 or more times. Based on this response, the respondents were further categorized as those with <3 or ≥3 vaccinations to represent those who did not get booster shots and those who got the booster shots. It is noteworthy that, although the recommended dosage of COVID-19 vaccine varies based on the type of vaccine, mRNA vaccines are offered in three primary dosages of 0.3 ml intramuscular, which include the initial dose (offered at 0 weeks), the second dose offered three weeks after the first dose, and the third dose administered eight weeks following the second dose.

Statistical analyses

Sampling weights were applied to account for the survey’s probability sampling design. Then, using a Pearson Chi-square test, the COVID-19 vaccination status of those who responded to the question “Ever been told you had COPD, emphysema, or chronic bronchitis?” was established with weighted proportions and counts reported. The same test was also used to determine the weighted proportion and count of those who received and those who did not receive COVID-19 vaccine boosters. With the identified group differences, a logistic regression analysis was conducted to establish the predictors of COVID-19 vaccination and receiving COVID-19 vaccination boosters using the characteristics that differed among the groups as the independent variables. Stata 14.0 (Stata Corp LLC, College Station, TX) was used for statistical analysis, and a two-tailed p-value <0.05 was considered statistically significant.

## Results

Among respondents with chronic pulmonary diseases, 1195/1486 (78.4%) had been vaccinated against COVID-19. Table 1 below shows the distribution of respondents with COPD by COVID-19 vaccination status. A larger proportion of individuals with chronic pulmonary disease were ≥65 years and most of them reported being vaccinated against COVID-19 [736 (87.0%) vs. 114 (13.0%), p<0.001]. Most were above the 1+ threshold for the ratio of family income to poverty, did not attend college [958 (76.9) vs. 260 (23.1), p<0.001], and were insured [1170 (79.7) vs. 270 (20.3%), p<0.001].

**Table 1:** COVID-19 vaccination status of respondents with COPD COPD: chronic obstructive pulmonary disease; DM: diabetes mellitus; NH: non-Hispanic

Characteristics		Total, n (%)	Unvaccinated [291 (21.6%)], n (%)	Vaccinated [1195 (78.4%)], n (%)	P-value
Age, years	<40	89 (100)	35 (42.9)	54 (57.1)	<0.001
	40 – 64	547 (100)	142 (26.1)	405 (73.9)	
	≥65	850 (100)	114 (13.0)	736 (87.0)	
Sex	Female	877 (100)	175 (21.3)	702 (78.7)	0.843
	Male	609 (100)	116 (21.9)	493 (78.1)	
Ethnicity	NH white	1163 (100)	236 (22.5)	927 (77.5)	0.320
	Hispanic	100 (100)	18 (18.5)	82 (81.5)	
	NH black	160 (100)	23 (15.2)	137 (84.8)	
	NH others	63 (100)	14 (26.2)	49 (73.8)	
Ratio to family income to poverty threshold	<1	316 (100)	102 (35.1)	214 (64.9)	<0.001
	≥1	1170 (100)	189 (18.1)	981 (81.9)	
College degree	No college	1218 (100)	260 (23.1)	958 (76.9)	<0.001
	College	261 (100)	27 (10.7)	234 (89.3)	
Insurance	Uninsured	46 (100)	21 (52.1)	25 (47.8)	<0.001
	Insured	1440 (100)	270 (20.3)	1170 (79.7)	
Obesity	Not obese	845 (100)	174 (22.9)	671 (77.1)	0.273
	Obese	607 (100)	108 (19.7)	499 (80.3)	
Smoking status	Never	372 (100)	58 (17.9)	314 (82.1)	<0.001
	Current	460 (100)	128 (30.0)	332 (70.0)	
	Former	630 (100)	97 (16.5)	533 (83.5)	
DM	No DM	1157 (100)	222 (21.7)	935 (78.3)	0.792
	DM	328 (100)	68 (20.9)	260 (79.1)	
Hypertension	No hypertension	530 (100)	118 (25.8)	412 (74.2)	0.018
	Have hypertension	956 (100)	173 (18.9)	783 (81.1)	

Table 2 below shows the distribution of respondents who received the COVID-19 boosters. Most were ≥65 years [545 (71.9) vs. 191 (28.1), p<0.001], NH whites [634 (65.8) vs. 293 (34.2), p=0.005], above the 1+ ratio to family income poverty ratio threshold [674 (64.9) vs. 307 (35.1), p=0.002], did not attend college [609 (60.6) vs. 349 (39.4), p=0.004], were insured [780 (63.3) vs. 390 (36.7), p=0.002], were former smokers [376 (67.0) vs. 157 (33.0), p=0.023], and were not obese [462 (65.9) vs. 209 (34.1), p=0.039].

**Table 2:** Distribution of respondents with COPD by COVID-19 vaccine booster status COPD: chronic obstructive pulmonary disease; COVID-19: coronavirus disease 2019; DM: diabetes mellitus; NH: non-Hispanic

Characteristics		Total, n (%)	Yet to receive boosters [406 (37.5%)], n (%)	Received boosters [789 (62.5%)], n (%)	P-value
Age, years	<40	54 (100)	32 (63.8)	22 (36.2)	<0.001
	40 – 64	405 (100)	183 (45.1)	222 (54.9)	
	65+	736 (100)	191 (28.1)	545 (71.9)	
Sex	Female	702 (100)	235 (28.6)	467 (61.4)	0.451
	Male	493 (100)	171 (36.1)	322 (63.9)	
Ethnicity	NH white	927 (100)	293 (34.2)	634 (65.8)	0.005
	Hispanic	82 (100)	33 (45.6)	49 (54.4)	
	NH black	137 (100)	60 (52.6)	77 (47.4)	
	NH others	49 (100)	20 (36.4)	29 (63.6)	
Ratio to family income to poverty threshold	<1	214 (100)	99 (49.4)	115 (50.6)	0.002
	≥1	981 (100)	307 (35.1)	674 (64.9)	
College degree	No college	958 (100)	349 (39.4)	609 (60.6)	0.004
	College	234 (100)	56 (27.4)	178 (72.6)	
Insurance	-	-	-		0.002
	Uninsured	25 (100)	16 (70.5)	9 (29.5)	
	Insured	1170 (100)	390 (36.7)	780 (63.3)	
Obesity	Not obese	671 (100)	209 (34.1)	462 (65.9)	0.039
	Obese	499 (100)	188 (41.6)	311 (58.7)	
Smoking status	Never	314 (100)	100 (37.0)	214 (63.0)	0.023
	Current	332 (100)	144 (44.6)	188 (55.4)	
	Former	533 (100)	157 (33.0)	376 (67.0)	
DM	No DM	935 (100)	323 (39.2)	612 (60.8)	0.057
	DM	260 (100)	83 (32.3)	177 (68.7)	
Hypertension	No hypertension	412 (100)	159 (41.8)	253 (58.2)	0.065
	Have hypertension	783 (100)	247 (35.0)	536 (65.0)	

As shown in Table 3 below, the positive predictors of COVID-19 vaccination were as follows: age 40 - 64 years (OR: 2.34, 95% CI: 1.31 - 4.19; p=0.004) and ≥65 years (OR: 1.93, 95% CI: 1.36 - 2.72; p<0.001), ratio to family income to poverty threshold ≥1 (OR: 2.02, 95% CI: 1.42 - 2.88; p<0.001), having a college degree (OR: 1.92, 95% CI: 1.92 - 3.26, p=0.016), and being insured (OR: 3.12, 95% CI: 1.46 - 6.66, p=0.003). The only negative predictor of COVID-19 vaccination was the current use of cigarettes (OR: 0.54, 95% CI: 0.33 - 0.87, p=0.012).

**Table 3:** Predictors of COVID-19 vaccination in patients with coronary heart disease CI: confidence interval; COVID-19: coronavirus disease 2019; Ref: reference group

Characteristics	Odds ratio (95% CI)	P-value
Age, years		
<40	Ref	
40 – 64	2.34 (1.31 – 4.19)	0.004
≥65	1.93 (1.36 – 2.72)	<0.001
Ratio of family income to poverty threshold <1	Ref	
≥1	2.02 (1.42 – 2.88)	<0.001
College degree	-	
No college	Ref	
College	1.92 (1.13 – 3.26)	0.016
Insurance	-	0.003
Uninsured	Ref	
Insured	3.12 (1.46 – 6.66))	
Smoking status	-	
Never	Ref	
Current	0.54 (0.33 – 0.87)	0.012
Former	0.81 (0.51 – 1.30)	0.390
Hypertension	-	0.417
No hypertension	Ref	
Have hypertension	1.16 (0.81 – 1.66)	

As shown in Table 4, the positive predictors of COVID-19 vaccination boosters included age 40 - 64 years (OR: 2.72, 95% CI: 1.39 - 5.30, p=0.003) and ≥65 years (OR: 4.85, 95% CI: 2.45 - 9.58, p<0.001). Being an NH black was a negative predictor of receiving the COVID-19 booster (OR: 0.55, 95% CI: 0.36 - 0.85, p=0.007).

**Table 4:** Predictors of COVID-19 vaccination boosters in patients with COPD CI: confidence interval; COPD: chronic obstructive pulmonary disease; COVID-19: coronavirus disease 2019; NH: non-Hispanic; Ref: reference group

Characteristics		Odds ratio (95% CI)	P-value
Age, years	<40		
	40 – 64	Ref 2.72 (1.39 – 5.30)	0.003
	≥65	4.85 (2.45 – 9.58)	<0.001
Ethnicity	NH white	Ref 0.60 (0.34 – 1.10)	0.100
	Hispanic	0.55 (0.36 – 0.85)	0.007
	NH black	1.03 (0.45 – 2.36)	0.941
	NH others		
Ratio to family income to poverty threshold <1, ≥1		Ref 1.49 (0.97 – 2.24)	0.080
College degree	No college		
	College	Ref 1.46 (0.96 – 2.24)	0.080
Insurance	Uninsured		
	Insured	Ref 1.99 (0.80 – 4.96)	0.138
Obesity	Not obese		
	Obese	Ref 0.80 (0.59 – 1.08)	0.150
Smoking status	Never		
	Current	Ref 0.70 (0.46 – 1.06)	0.088
	Former	0.91 (0.61 – 1.35)	0.629

## Discussion

It is estimated that 16 million adults living in the US have COPD, and more than half of the diagnosed patients are women [11]. Our study revealed that almost four in five adults (78.4%) with COPD have been vaccinated against COVID-19, and about three in five (62.5%) have received booster vaccines. These individuals who have been vaccinated and received booster shots against COVID-19 were mostly aged ≥65 years. Factors predicting higher vaccination include older age and a better socioeconomic status, the indicators of which include being insured, having a college degree, and being above the ratio of family income to the poverty threshold of 1+. Negative predictors of receiving COVID-19 vaccine boosters include current cigarette use and being a non-Hispanic Black individual.

Although the findings related to vaccination rates in this population are commendable, these are inferior to the COVID-19 vaccination rate reported in a group of COPD patients in Hungary (86.5%) [11]. This difference may be due to the smaller sample size of the latter study and because it was conducted at the peak of the COVID-19 pandemic. In contrast, a lower vaccination rate of 39.0% was reported in an outpatient COPD population in Beijing [14], a city with a smaller population and lower COVID-19-related death rate as compared to the US. Thus, the observed COVID-19 rate in our study may be related to the higher COVID-19 death rates in the US as compared to other countries [15-21] and the consequent high vaccine awareness and uptake campaigns in the US.

One striking revelation pertains to age as a determinant for COVID-19 vaccination. Our study results unequivocally demonstrate that individuals aged 40 years and above exhibited a markedly higher likelihood of following the primary vaccine recommendations against COVID-19 and the subsequent recommendations about booster doses. These findings have been endorsed by a review article that reported older age, among other variables, as an important factor in vaccination uptake [16-21]; moreover, other studies on the topic have various limitations.

Our study employed a mediation framework to explain the racial and ethnic disparities in COVID-19 vaccine uptake with a national longitudinal survey. Other studies have also reported similar associations between older age and vaccination uptake [8,22]. This age bracket, often associated with increased vulnerability to severe outcomes from the virus, showed a more proactive approach towards vaccination. This age group's proclivity to get vaccinated and opt for booster doses suggests a keen awareness of the necessity for added protection.

Socioeconomic status emerged as a pivotal factor influencing vaccination decisions. The correlation between higher vaccination rates and a better financial standing, denoted by a higher income threshold, highlights the influence of economic stability on accessing and embracing vaccination opportunities. In addition, having health insurance, often indicative of improved healthcare access, also plays a significant role, with insured individuals displaying higher vaccination rates. Previous studies on predictors of COVID-19 vaccination among the general population have shown that both the levels of education and income exhibit positive relationships with COVID-19 vaccine uptake with an inverse relationship reported with financial hardship [16], a finding endorsed by Williams et al. [17]. In addition, they also found a negative association between racial minority groups and COVID-19 vaccination [17], not with primary vaccination but with receiving booster shots.

However, the picture is not entirely uniform across all socioeconomic markers. An intriguing observation related to educational attainment. In contrast with a previous study [9], a lack of college attendance was associated with higher vaccination rates. In general, mixed findings regarding this relationship have been reported, with one study reporting both negative and positive associations between educational attainment and COVID-19 vaccine uptake [18]. This incongruity challenges preconceived notions and urges a more nuanced understanding of the diverse factors shaping vaccination behaviors within different educational strata. Nevertheless, these findings are worrying because US populations with chronic pulmonary diseases are more likely to belong to a low socioeconomic class [19] and suffer from more severe chronic pulmonary disease or COVID-19-specific outcomes [20]; thus, a higher socioeconomic profile driving vaccination rates could indicate a further worsening of health outcome disparities in this population.

Predictors for COVID-19 vaccination among adults with chronic pulmonary diseases extend beyond age and socioeconomic status. Our results underscore the influence of individual health behaviors, with current cigarette use emerging as a negative predictor of COVID-19 vaccination. Studies have reported mixed findings about this relationship in populations without chronic pulmonary diseases, with tobacco use reportedly associated with low uptake of the COVID-19 vaccines in one study [23] but, in contrast, was not associated with lower uptake in another study [24]. Furthermore, a specific racial demographic, the NH black race in this case, was associated with a lower likelihood of receiving booster shots. The reported hesitancy and resistance in the intention to adopt the COVID-19 vaccine by NH blacks can be attributed to the lack of trust, among various factors, towards the vaccine manufacturers and the government. Thus, mistrust in the extant healthcare system alongside concerns regarding the effectiveness of the vaccines and their safety are among the notable factors underlying the lower likelihood of adopting COVID-19 vaccines by NH blacks. These findings not only elucidate the intricate relationship between lifestyle choices and health-related decision-making during a pandemic but also call attention to the existence of systemic or cultural factors that warrant deeper exploration. Thus, targeted strategies are needed to address and mitigate barriers to booster uptake among marginalized communities and populations with health behaviors associated with a lower vaccination rate.

Strengths and limitations

This study has some strengths and limitations worthy of mention. The study population comprised US adults with COPD, and as such, with the application of survey weights in the analysis, the estimates are nationally representative. The use of survey weights also meant that we had a large sample, and as such, the study was powered to answer the question it set out to answer. However, despite these strengths, given that this is a non-experimental study, the predictors of COVID-19 vaccination observed should be analyzed with caution as true causation is difficult to prove with these kinds of studies. Given that the data were collected using surveys, recall bias among the study participants is a possibility, a problem that cannot be corrected or addressed in these kinds of studies. Additionally, the other major limitation pertains to the observation that Native Americans have lower rates of vaccination adoption in comparison to other minority groups, including NH blacks; however, in this study, they were not aptly acknowledged as a distinctive group and this may be attributed to the smaller sample size. Also, for this study, the dates of vaccination were self-reported, which might impact the accuracy of dates. Lastly, given that the study explored the ethnic and racial disparities concerning vaccine hesitancy and adoption, the failure to aptly explore the mediating mechanisms might lead to the perpetuation of dangerous misunderstandings and myths, which might negatively impact health promotion efforts.

## Conclusions

Our findings highlight the multifaceted nature of vaccination-related decision-making, which underscores the need for tailored public health strategies accounting for the diverse socioeconomic, behavioral, and demographic characteristics among adult populations with COPD. Acknowledging these intricacies will be pivotal in formulating inclusive and effective interventions to ensure equitable access to vaccination and boosters, thereby mitigating the impact of COVID-19 in this vulnerable group. Among the notable recommended strategies to enhance vaccine adoption include the strengthening of efforts to enhance vaccine confidence in expectation of prospective updated COVID-19 vaccines, as this is likely to improve adoption, particularly in populations with higher rates of hesitancy. Moreover, the prospective public health strategies and efforts should focus on younger individuals, ethnic and racial minority groups, rural residents, and individuals residing in socially vulnerable regions, as they are more likely to gain from the public health outreach efforts in addition to realizing timely completion of COVID-19 vaccination.

Though age has emerged as a consistent aspect in the research on this topic, various socioeconomic determinants such as education, income level, and insurance status are also likely to impact vaccine adoption rates. As such, new strategies must be developed to create a greater level of awareness of the importance of vaccination, particularly in minority populations. Lastly, further research is needed to enhance public health strategies in addition to ensuring that vulnerable populations have increased and fair access to vaccinations.
